# Seven Years' Working of the Midwives Act

**Published:** 1909-04-10

**Authors:** 


### Nursing Administration.

### SEVEN YEARS' WORKING OF THE MIDWIVES ACT.

The recently issued report of the Central Midwives Board presents in highly condensed form the result of the vigorous efforts which have been in progress for the last seven years to convert the untrained midwives of this country into skilled officers, amenable to discipline and under the complete control of the county authorities. In many respects the report is admirable. It gives but a bare record of the work accomplished, but between the lines it is easy to read the immense amount of personal zeal and ability which has been enlisted on behalf of the mothers and the unborn generation. The members of the Board had to construct their own precedents, interpret the laws governing their actions, frame their own rules, and establish a standard which should not on the one hand discourage the unlearned, and should yet justify their own existence. They have done much. A year ago the number of midwives on the roll was 25,634, of whom 3,326 have been admitted since the expiry of the period of grace. But of this number about half were either not practising midwifery or were doing so without notifying to the local supervising authority. An unknown number had died without their demise being reported to the Board, only 324 deaths having been officially notified during the existence of the roll. In addition to the women whose names are entered on the roll, a large number of uncertified women are known to be continuing their practice. The Midwives Act, then, has so far but imperfectly fulfilled its object of gathering together all the midwives in the country under, and placing them under, inspection. Nearly half of the women whose names they have collected are under no inspection. And an unknown number of women whose names have never been entered are continuing their calling. But this is not all. The figures indisputably show that the Act, although it is having a favourable effect in stimulating the study of midwifery and in repressing some of the worst abuses is not having the desired effect in replacing the old type of midwife by trained women. True the old type is dying out fast, but she is not being replaced. And the examinations of the Central Midwives Board are not attracting the women who could replace them. The 71 recognised training schools, the 88 recognised teachers, and the 92 approved midwives who are engaged in the instruction of the future midwife are unfortunately far too busy teaching midwifery to persons who regard this branch of work as an interesting asset in some other walk of life to trouble about the women who are fitted to live the life of the people and take upon them the duties of the moribund midwife of the old style. The total number of successful candidates in examinations held by the Board in the three years since they were instituted is 4,045; "but," says the Report, "less than 60 per cent. of the successful candidates intend to practise as midwives, the net annual addition of practising midwives to the roll being at present under 1,000." About 10 per cent. of nurses in the prime of life in good work are lost to the profession from various causes every year. There can be little doubt that out of the 15,000 practising midwives, certified and uncertified, who it is stated in this Report are probably in practice at the present time, an annual wastage takes place of at least 15 per cent., for many are elderly women. Thus, while over 2,000 midwives fall out of the ranks for one reason or another every year, the annual number of new midwives enrolling themselves with the intention of practising as midwives is under a thousand. But this is not the most serious part of the matter. If it were only that the midwives needed by working mothers were not yet being provided in sufficient numbers to meet the demand, it would all come right in time. But when out of the successful candidates in the examinations nearly half do not intend to practise midwifery at all, and out of those who do intend to practise an overwhelming majority are not intending to practise among the poor, but are using their certificate as an additional qualification in the practice of monthly nursing among the rich, it is time to inquire in whose interests the regulations responsible for this condition of affairs have been framed. Is it a healthy thing for the public and for the midwives that every encouragement should be given to the creation of training schools for midwives in institutions, while every possible discouragement is shown to instruction being given throughout the country in the homes of the people? The Report says quite justly that "the Board is not authorised by the Midwives Act to undertake the training of midwives," but it also observes that an important part of the work lies in the authorisation of persons through whom this instruction may be given. Unfortunately the regulations have been framed with a view to this instruction being given exclusively in institutions. Now the system of training in institutions cannot be developed further without a large increase in the number of mothers who resort to institutional aid, whether meted out by private charity or by the Poor Law, in child-bearing. We do not believe this development to be in the best interests of motherhood. The instinct which leads a mother to prefer her own home for the natural process of child-bearing is a wholesome one, and ought to be encouraged. When we find—as we do from this Report—that, so far from fitting out a well-trained body of women for service among the class who rely upon the ministrations of midwives, the institution training schools are chiefly engaged in bestowing a superfluous qualification on people who never intend to make regular use of their skill, it becomes evident that the rules and regulations of the Midwives Act are failing in the purpose for which they were framed; and as long as this is so, the shortage of practical midwives will grow steadily more serious every year.

